# Identification of natural killer cell associated subtyping and gene signature to predict prognosis and drug sensitivity of lung adenocarcinoma

**DOI:** 10.3389/fgene.2023.1156230

**Published:** 2023-04-07

**Authors:** Dexin Zhang, Yujie Zhao

**Affiliations:** ^1^ Respiratory Department of the Second Affiliated Hospital of Xi’an Jiaotong University Medical College, Xi’an, China; ^2^ Regional Marketing Department, Yuce Biotechnology Co, Ltd., Dabaihui Center, Shenzhen, China

**Keywords:** lung adenocarcinoma, NK cells, programmed cell death, immunity, survival, prediction model, chemotherapy drug

## Abstract

**Introduction:** This research explored the immune characteristics of natural killer (NK) cells in lung adenocarcinoma (LUAD) and their predictive role on patient survival and immunotherapy response.

**Material and methods:** Molecular subtyping of LUAD samples was performed by evaluating NK cell-associated pathways and genes in The Cancer Genome Atlas (TCGA) dataset using consistent clustering. 12 programmed cell death (PCD) patterns were acquired from previous study. Riskscore prognostic models were constructed using Least absolute shrinkage and selection operator (Lasso) and Cox regression. The model stability was validated in Gene Expression Omnibus database (GEO).

**Results:** We classified LUAD into three different molecular subgroups based on NK cell-related genes, with the worst prognosis in C1 patients and the optimal in C3. Homologous Recombination Defects, purity and ploidy, TMB, LOH, Aneuploidy Score, were the most high-expressed in C1 and the least expressed in C3. ImmuneScore was the highest in C3 type, suggesting greater immune infiltration in C3 subtype. C1 subtypes had higher TIDE scores, indicating that C1 subtypes may benefit less from immunotherapy. Generally, C3 subtype presented highest PCD patterns scores. With four genes, ANLN, FAM83A, RHOV and PARP15, we constructed a LUAD risk prediction model with significant differences in immune cell composition, cell cycle related pathways between the two risk groups. Samples in C1 and high group were more sensitive to chemotherapy drug. The score of PCD were differences in high- and low-groups. Finally, we combined Riskscore and clinical features to improve the performance of the prediction model, and the calibration curve and decision curve verified that the great robustness of the model.

**Conclusion:** We identified three stable molecular subtypes of LUAD and constructed a prognostic model based on NK cell-related genes, maybe have a greater potential for application in predicting immunotherapy response and patient prognosis.

## 1 Background

Lung cancer is a leading cause of cancer mortality in the world (Hirsch et al., 2017). Statistics reported that in 2022 in the United States will die from cancer, and approximately 350 of them die from lung cancer every day (Siegel et al., 2022). Adenocarcinoma (lung adenocarcinoma, LUAD) is currently the predominant histologic type, which accounts for approximately 50% of all lung cancer cases, and is notable for its high incidence, high mortality, and poor prognosis (Succony et al., 2021). Currently, surgery is recommended for early-stage lung cancer and is considered the most effective treatment option, while those with advanced disease are often further supplemented with radiotherapy, chemotherapy, targeted therapy, and immunotherapy (Hoy et al., 2019). Regardless of the interventions used, the overall 5-year survival of LUAD patients remains below 20% (Duma et al., 2019). Therefore, it is necessary to develop current understanding on the pathogenesis of LUAD to provide a theoretical basis for reducing the occurrence of LUAD, improving the treatment of LUAD and its prognosis.

The development of LUAD involves external environment, gene mutation, tumor immunity, and family genetics, and is a multistep, cascade process (Suster and Mino-Kenudson, 2020). As a component of the tumor microenvironment, tumor immune cells are present in all stages of LUAD and play an important role in shaping tumor development (Saab et al., 2020). For example, tumor-associated macrophages can accelerate tumor progression by promoting tumor angiogenesis, metastasis and immune escape. Regulatory T cells inhibit anti-tumor immune responses, thereby promoting the development of immunosuppressive tumor microenvironments and promoting cancer progression (Hsieh et al., 2012). Cytotoxic CD8^+^ memory T cells kill tumor cells by recognizing specific antigens on them and stimulating an immune response (Arneth, 2019). Dendritic cells are antigen-presenting cells, which are an important bridge between innate and adaptive immunity. Dendritic cells can not only induce cellular immunity and humoral immunity, but also activate natural killer (NK) cells and NK T cells (Sadeghzadeh et al., 2020). NK cells are anti-tumor immune cells that kill cancer cells in the body, but in the tumor microenvironment NK cells are generally reduced in number and impaired in function (Russell et al., 2022). Basic experiments and clinical studies together have shown that NK cells are in the first line of defense against tumors and do not require pre-stimulation to cause NK cells to migrate to the lesion and play an immunomodulatory role (Guillerey, 2020). Phenotypically, NK cell subpopulations display potent antitumor immune cytotoxicity *via* MEK/ERK and PI3K/Akt/mTOR pathways upon stimulation through cytokines such as interleukin (IL) (Valipour et al., 2019). Although patient’s immune system can recognize neoantigens produced by tumors with high mutational load (immunogenic “hot” tumors), in terms of its mutational load, lung cancer is immunogenic, only moderately, to some extent. Therefore, the highly complex interaction between LUAD and NK cells is a major challenge to improve immunotherapy.

Studies on the pathogenesis of NK cells in LUAD have delved into the genetic-molecular field, and it is mostly believed that the development of LUAD is the result of a multigene, multistage involvement (Crinier et al., 2020). However, the genetic landscape and immune profile of NK cells in LUAD are unclear, also the prognosis and immune efficacy prediction of LUAD based on NK cells have not been reported. This study first identified stable molecular subtypes of LUAD by consistent clustering of NK cell-associated genes, and further compared clinicopathological, mutational, immunological, and pathway characteristics among the subtypes. Then, we constructed a risk model and a clinical prognostic model, which can be used to evaluate personalized treatment for LUAD patients.

## 2 Materials and methods

### 2.1 Source of clinical information and gene expression profile data of NK cells

The clinical information and mRNA transcriptome data of LUAD patients were downloaded from the TCGA GDC API (Colaprico et al., 2016). To verify the accuracy of the results, we also downloaded the clinical and mRNA gene expression data of LUAD patients from the Gene Expression Omnibus database (GEO) database (Toro-Domínguez et al., 2019), including GSE72094, GSE31210 datasets. The TCGA dataset contained 500 LUAD samples as the training set, while the GSE72094 and GSE31210 datasets contained 398 and 226 LUAD samples, respectively, as the validation set.

To ensure the quality and reliability of the downloaded data, quality control was performed, and the inclusion and exclusion criteria were (Hirsch et al., 2017) to remove samples with incomplete clinical information; (Siegel et al., 2022); to remove samples with unknown survival time and survival status; (Succony et al., 2021); to remove probes with one probe matching to multiple genes, and the mean value was taken as the expression value of that gene when multiple probes matched to one gene.

NK cell-associated genes were obtained from three parts, including the ImmPort official website (https://www.immport.org/resource), the MSigDB database (Molecular Signatures Database, https://ngdc.cncb.ac.cn/databasecommons/database/id/1077) and the LM22 database (Newman et al., 2015), containing 134 cell-associated genes, 18 NK cell-associated pathways, and 79 NK cell-associated genes, respectively.

### 2.2 Subtyping of LUAD patients based on NK cell-associated genes

A total of 213 NK cell-associated genes and 18 NK cell-associated pathways were obtained from the three databases, and we used the single sample gene set enrichment analysis (ssGSEA) method to evaluate these 213 NK cell-associated genes and 18 NK cell-associated pathways in the TCGA and GEO datasets, respectively. The samples were then clustered by ConsensusClusterPlus using these pathway scores in the TCGA and GEO cohorts, and the “K-M” algorithm and “1-Pearson correlation” as the metric distance (Azman et al., 2006). We conducted 500 bootstraps, with each one including 80% patients of in the training set and 20% those of the validation set. Finally, based on the cumulative distribution function (CDF), the optimal number clusters were decided, and the optimal classification and the sample molecular subtyping was obtained by calculating the consistency matrix and the consistency cumulative distribution function (Zhang et al., 2021a).

### 2.3 Immunological features and pathway analysis among different molecular subtypes

We obtained the molecular characteristics of LUAD genomic alterations from published literature, including LOH, Aneuploidy Score, tumor mutation burden (TMB), purity, and ploidy, Homologous Recombination Defects, Intratumor heterogeneity. The relative abundance of 22 immune cells were calculated using CIBERSORT R package. At the same time, we used the ESTIMATE algorithm R package to calculate the proportion of immune cells and finally compared the inflammatory and immune activity scores (Chakraborty and Hossain, 2018; Chen et al., 2018).

We performed gene set enrichment analysis (GSEA) on all NK cell-associated genes in the Hallmark database, and then used the ssGSEA method to calculate the pathway scores for both TCGA and GEO datasets in the GSVA package (Barbie et al., 2009). A false discovery rate (FDR) of <0.05 in this study was considered statistically significant.

### 2.4 Drug sensitivity analysis between molecular subtypes

Immune checkpoint inhibitor (ICI)-based therapy has become one of the standard treatments for advanced lung cancer (Zhang et al., 2021b). We first assessed the expression of genes associated with immunotherapy, such as CTLA4, PD-L1, and PD-1, among various molecular subtypes to determine whether there were differences in immunotherapy responsiveness among them. Next, we used the TIDE software (http://tide.dfci.harvard.edu/) to assess the potential clinical effects of immunotherapy in our defined molecular subtypes. Greater likelihood of immune escape was correlated with a higher TIDE prediction score, suggesting that patients may benefit less from immunotherapy (Jiang et al., 2018). Finally, we performed drug sensitivity prediction for LUAD in the “pRRophetic” package (Geeleher et al., 2014).

### 2.5 Identification of key NK cell-related genes among molecular subtypes

The differentially expressed genes among different molecular typing were calculated by the “limma” package, using FDR <0.05 and |log2FC| > 1 as the statistical difference criteria, and visualized the differentially expressed genes by “pheatmap” and “ggplot2” R packages in a heatmap and volcano map. Then, all genes with statistically significant differences were enriched using the “clusterProfiler” package.

Next, we performed univariate Cox regression analysis for differentially expressed genes between molecular subtypes, and then reduced the prognosis-related genes by Lasso regression (Sun et al., 2021), which can better solve the problem of multicollinearity in regression analysis by compressing some coefficients and setting some coefficients to zero at the same time. With the gradual increase of lambda, we selected the number of factors when the coefficients of independent variables tended to zero. Then, we used the AIC deficit pool information criterion through stepwise regression, which has the advantage of considering the statistical fit of the model and the number of parameters used to fit it, and at the same time indicates a sufficient fit of the model obtains with fewer parameters (Zhang, 2016).

### 2.6 Construction and validation of the prognostic model

We calculated the NK cell-related prognostic RiskScore for each sample according to the formula defined by the sample RiskScore (below), and normalized it (Nie et al., 2021).

RiskScore=coefficient1*gene1 expression+…+coefficientN*geneN expression.

After that, LUAD patients were divided into high- and low-risk groups based on the relationship between RiskScore and 0, where those with RiskScore >0 were considered as having a high risk and those with RiskScore <0 were considered as having a low risk. Finally, the survival differences between the two groups were compared by log-rank test. In order to verify the robustness of the model, we performed immune signature analysis, survival curve, and drug treatment difference analysis for the patients in the two groups.

### 2.7 Improvement of prognostic models and survival prediction in LUAD patients

To more accurately quantify the risk assessment and survival probability of LUAD patients, we combined the RiskScore with other clinicopathological characteristics of LUAD patients and constructed a nomogram using the “nomogramEx” R package. To validate the accuracy of the model, a calibration curve was plotted by the “PredictABEL” function to visualize the goodness-of-fit. This was followed by decision curve analysis (DCA) to describe the change in net benefit as the threshold probability changed under the intervention of the predicted value by the model (Van Calster et al., 2016; Van Calster et al., 2018).

### 2.8 Programmed cell death (PCD) analysis

12 PCD patterns (apoptosis, necroptosis, pyroptosis, ferroptosis, cuproptosis, entotic cell death, netotic cell death, parthanatos, lysosome-dependent cell death, autophagy-dependent cell death, alkaliptosis, and oxeiptosis) have been taken from the previous research (Zou et al., 2022). ssGSEA analysis based on the expression data of PCD related genes using the R package GSVA. Spearman analysis was conducted to know the relationship among PCD patterns, clinical features, RiskScore in LUAD samples.

### 2.9 Statistical analysis

Unless otherwise specified, all statistical tests were bilateral and conducted using R software (version 4.1.3, https://www.r-project.org/), and *p* < 0.05 was considered statistically significant.

## 3 Results

### 3.1 Molecular subtyping of LUAD based on NK cell-associated genes

**FIGURE 1:**
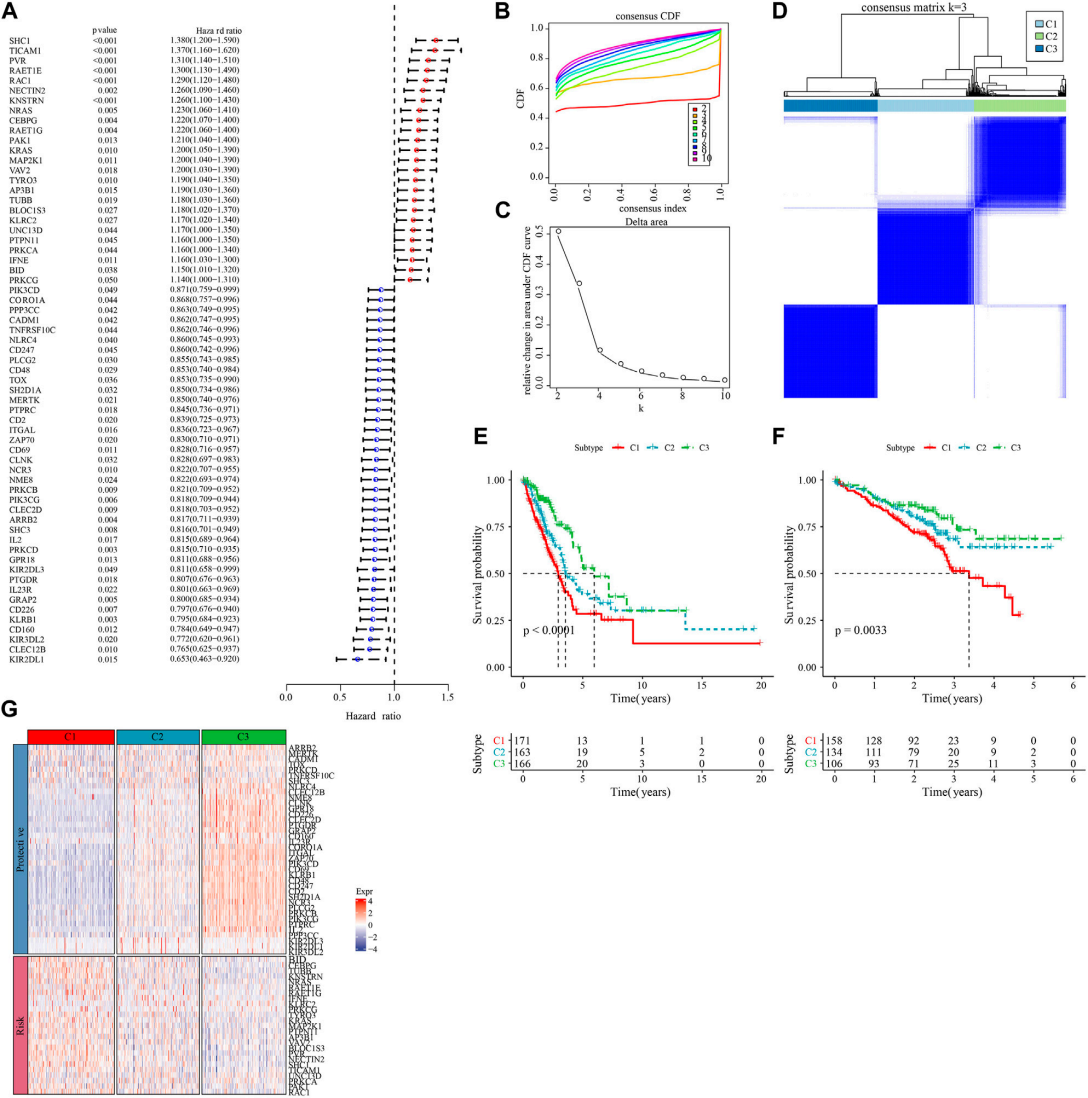
Molecular subtyping based on natural killer cell-associated genes. **(A)** Forest plot of prognostically significant natural killer cell-associated genes in the TCGA-LUAD cohort. **(B)** CDF curves of the TCGA-LUAD cohort. **(C)** CDF Delta area curves of the TCGA-LUAD cohort. **(D)** heat map of sample clustering at consensus *k* = 3 in the TCGA-LUAD cohort. **(E)** KM curves of the relationship between overall survival (OS) prognosis of the three subtypes in the TCGA-LUAD cohort. **(F)** Prognostic differences between the three molecular subtypes in the GSE72094 cohort. **(G)** Heatmap of prognosis significant natural killer cell genes expression in different subtypes of TCGA-LUAD.

We first calculated the NK cell-related genes showing close relationship with LUAD survival chance by univariate Cox regression analysis, and screened 63 prognostically significant genes (*p* < 0.05, Figure 1A), including the prognostic (Protective) genes SHC1, TICAM1, PVR, RAET1E, RAC1 (HR > 1), and KLRB1, CD160, KIR3DL2, CLEC12B, and KIR2DL1 (HR < 1). Then, we used these 63 differential genes for consistent clustering, and determined the best cluster number according to CDF. And we could see from Figures 1B, C that Cluster = 3 had more stable clustering results, hence, *k* = 3 was selected to obtain three molecular subtypes (C1, C2, and C3) (Figure 1D). Then, we performed survival analysis of patients with these three molecular subtypes using the K-M survival method, and the results identified a significant difference in prognosis among the three molecular subtypes, with C1 patients having the worst prognosis and C3 patients having the optimal prognosis (Figure 1E). The results were also validated in the GSE72094 dataset (Figure 1F). Meanwhile, we found that the “Risk” genes were high-expressed in the C1 subtype and the “Protective” genes were high-expressed in the C3 subtype in the heat map (Figure 1G). These results suggested that the molecular subtyping based on NK cell-related genes was reasonable, and there were significant differences in gene expression and prognosis among patients with different subtypes.

### 3.2 Genetic landscape between molecular subtypes of LUAD

**FIGURE 2:**
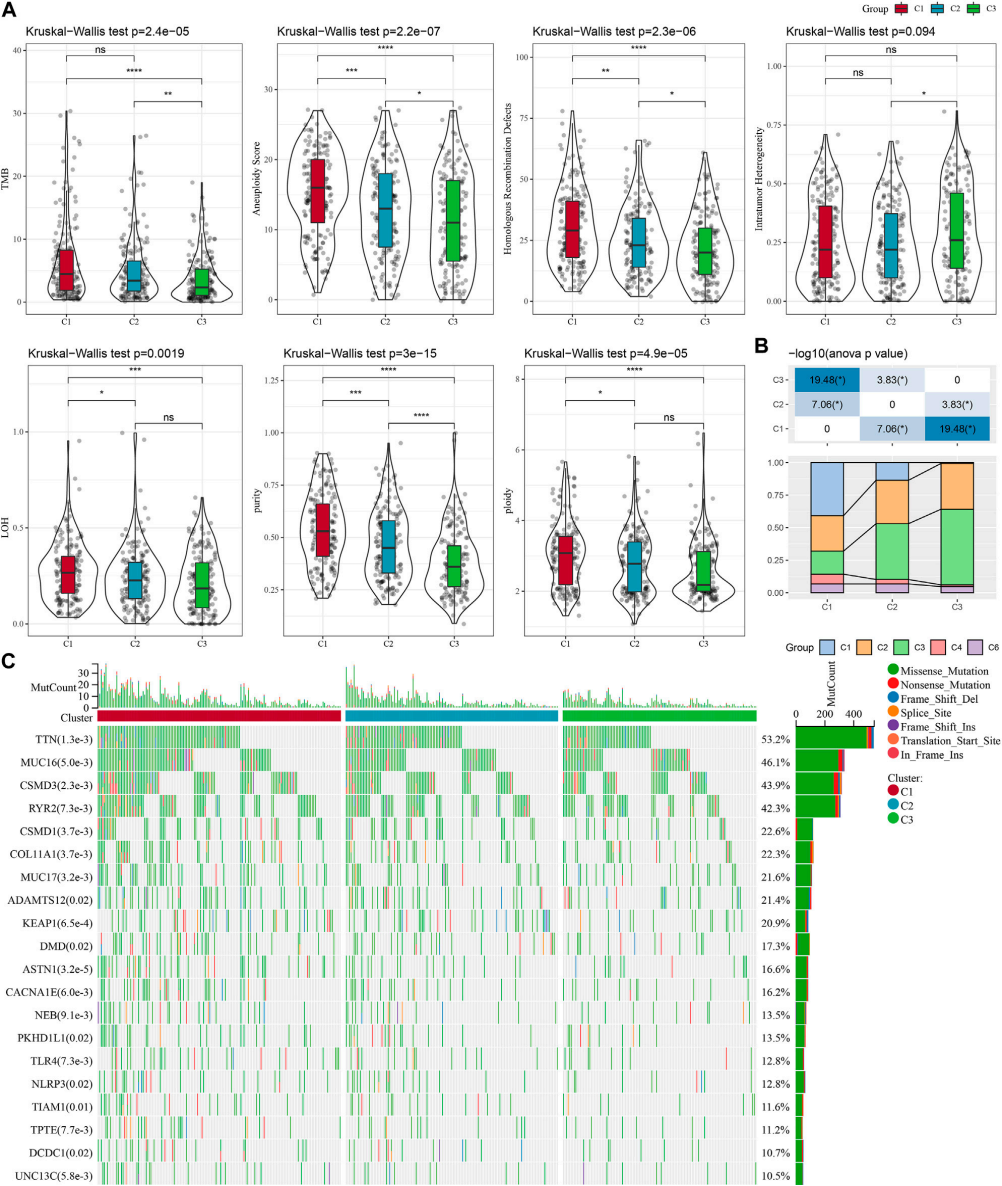
Genomic alterations in the molecular subtypes of the TCGA cohort. **(A)** Comparison of differences in Homologous Recombination Defects, Aneuploidy Score, Fraction Altered, Number of Segments, and Tumor mutation burden in the TCGA cohort molecular subtypes. **(B)** Comparison of the three molecular subtypes with immune molecular subtypes. **(C)** Somatic mutations in the three molecular subtypes (chi-square test). *p < 0.05; **p < 0.01; ***p < 0.001; ****p < 0.0001.

To explore the differences in specific gene expression profiles among different molecular subtypes, we compared the molecular profiles among C1, C2, and C3 subtypes of LUAD samples, respectively, and it is obvious from Figure 2A that purity, and ploidy, TMB, Aneuploidy Score, LOH, Homologous Recombination Defects expression were the highest in C1 but the lowest in C3, which was consistent with previous studies (Thorsson et al., 2018). In addition, we compared the differences between the molecular subtyping of published studies and that in this study. Here it was found that the C3 subclass occupied the most of the C3 subtypes we defined, suggesting that the C3 subtype was the major subtype of LUAD (Figure 2B). In addition, a significant correlation between molecular subtypes and gene mutations was detected after analyzing the correlation between gene mutations and molecular subtypes, and observed. TTN, MUC16, CSMD3, and RYR2 were the most widely mutated genes in LUAD (Figure 2C), and this finding indicated that the development of LUAD was closely related to the above-mentioned gene mutations.

### 3.3 Pathways enrichment analysis among the molecular subtyping of LUAD

**FIGURE 3:**
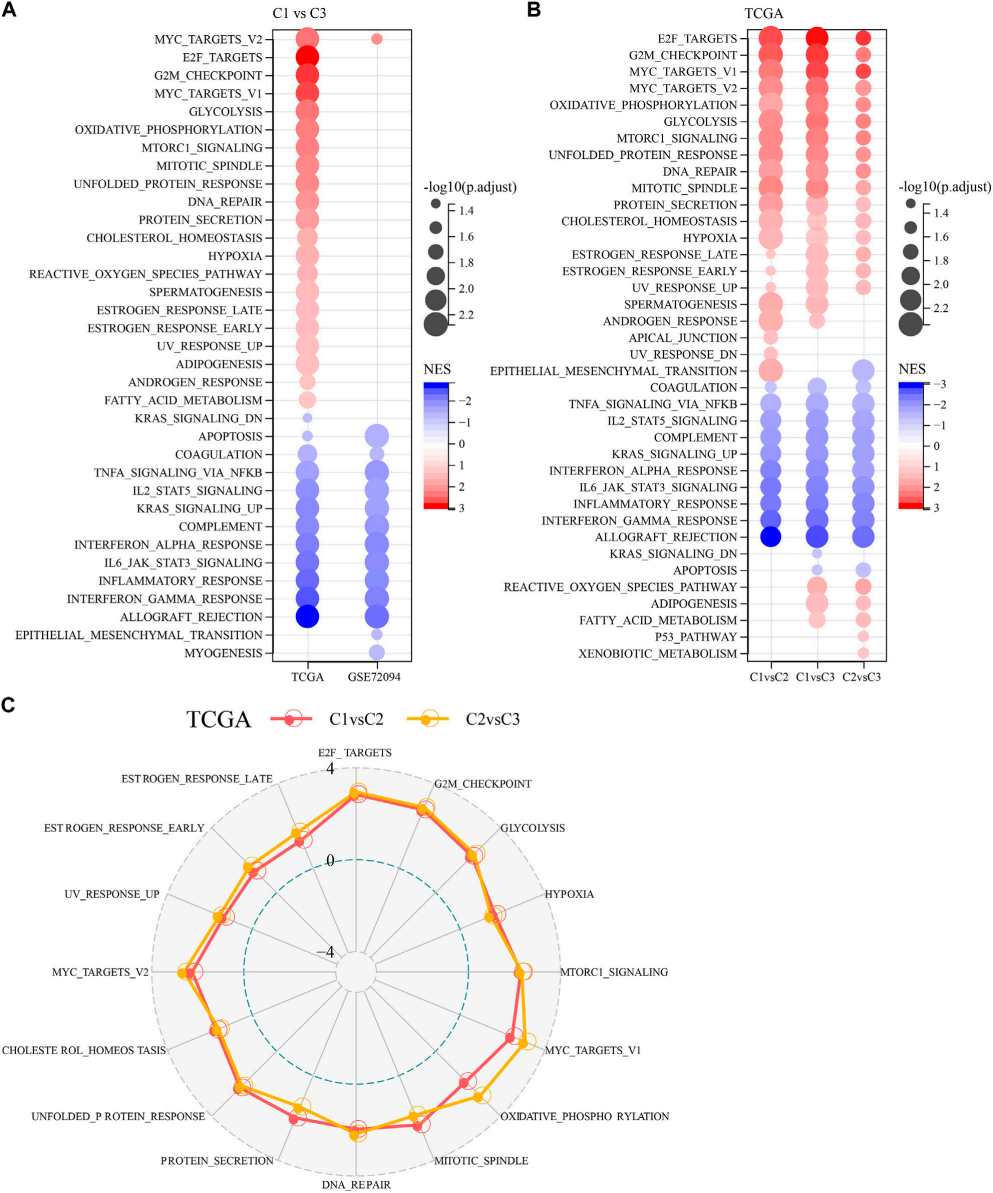
Pathway analysis between molecular subtypes. **(A)** Bubble plots of GSEA results for C1 vs. C3 subtypes in two lung adenocarcinoma cohorts. **(B)** Bubble plots of GSEA results for different molecular subtypes compared to each other in the TCGA-LUAD cohort. **(C)** Radar plots of C1 vs. C2 and C2 vs. C3 activation pathways in the TCGA-LUAD cohort.

To investigate pathway differences in LUAD among different molecular subtypes, we performed GSEA enrichment analysis among molecular subtypes. As shown in Figure 3A, we enriched a total of 33 significant pathways in the TCGA-LUAD dataset, including MYC_TARGETS_V2, E2F_TARGETS, NFLAMMATORY_ RESPONSE, MYOGENESIS, INTERFERON_GAMMA_RESPONSE, MYC_TARGETS_V1, GLYCOLYSIS, G2M_CHECKPOINT, EPITHELIAL_MESENCHYMAL_TRANSITION, ALLOGRAFT_REJECTION, suggesting that these NK cell genes were mainly associated with cell cycle and immunity in C1 and C3. Additionally, pathways different between C1 and C3 subtypes, between C2 and C3 subtypes, between C1 and C2, were analyzed (Figure 3B). Overall, the cell cycle pathway was activated in C1 patients, while the immune-related pathway was suppressed, therefore we hypothesized that these NK cell genes might play an important role in the cell cycle pathway as well as in the tumor microenvironment. To validate these results, we presented the pathway differences between C1 and C2, and C2 and C3 as radar plots, and the results showed that they both had significant consistency in cell cycle (MYC_TARGETS_V2, MTORC1_SIGNALING, MYC_TARGETS_V1) and immune-related pathways (G2M_CHECKPOINT, E2F_TARGETS, UNFOLDED_PROTEIN_ RESPONSE) (Figure 3C).

### 3.4 Immune characteristics among different molecular typologies of LUAD

**FIGURE 4:**
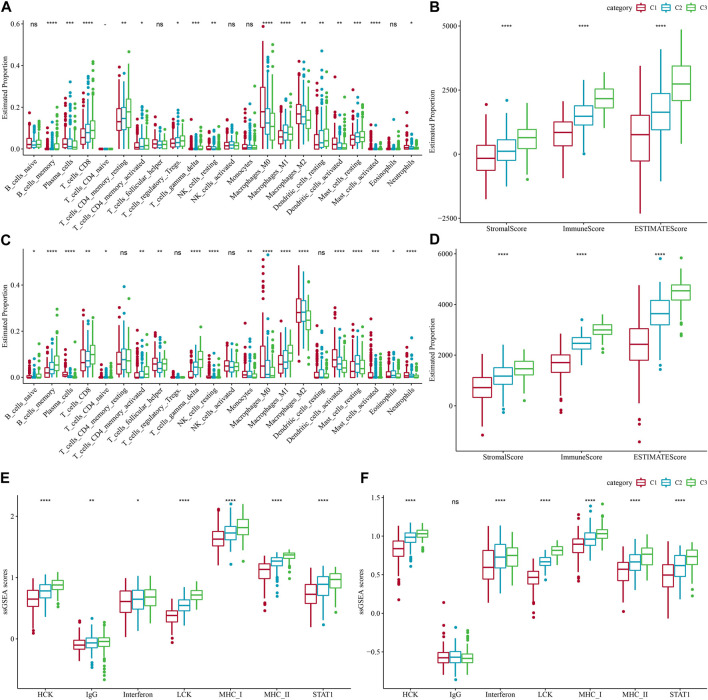
Proportions of immune cell components in the two lung adenocarcinoma cohorts. **(A)** Differences in 22 immune cell scores between different molecular subtypes in the TCGA-LUAD cohort. **(B)** Differences in ESTIMATE immune infiltration between different molecular subtypes in the TCGA-LUAD cohort. **(C)** Differences in the GSE72094 cohort 22 immune cell scores between different molecular subtypes. **(D)** Differences in ESTIMATE immune infiltration between different molecular subtypes in the GSE72094 cohort. **(E)** Differences in seven inflammation-related gene cluster scores across molecular subtypes in TCGA-LUAD cohort. **(F)** Differences in gene cluster scores between different molecular subtypes in seven inflammatory-related genes in GSE72094 cohort.

The immune system plays a dual role in the development of LUAD, as it can recognize and destroy tumor cells, while tumor cells can also evade host immune attack by forming a complex immunosuppressive network under the pressure of immune selection using the immune system’s own negative regulatory mechanisms, thus the TME is in a constant state of change (Anichini et al., 2020; Spella and Stathopoulos, 2021). To explore the immune landscape among different molecular subtypes of LUAD, we first assessed the differences in the components of immune cells in the TCGA-LUAD cohort using the CIBERSORT algorithm and observed that most immune cells (B cells, T cells, NK cells, *etc.*) were significantly different (*p* < 0.05) (Figure 4A). We then used the ESTIMATE algorithm to assess immune cell infiltration, and the results showed that StromalScore, ImmuneScore and EstimateScore were significantly different between C1, C2, and C3 (*p* < 0.05)), with ImmuneScore accounting for the largest proportion of C3 types, suggesting a higher degree of immune infiltration in C3 subtypes (Figure 4B). Similarly, we obtained results in the GSE72094-LUAD cohort that were consistent with the TCGA-LUAD cohort (Figures 4C, D). In addition, we assessed the inflammatory activity of C3, C2, C1, except for IgG, the remaining six out of 7 metagenes clusters (HCK, Interferon, LCK, MCH I, MCH II, and STAT1) showed significantly different enrichment scores, with the C4 subtype having higher inflammatory activity (Figure 4E). The findings were consistent in the GSE72094-LUAD cohort (Figure 4F).

### 3.5 Differences in immunotherapy between molecular subtypes

**FIGURE 5:**
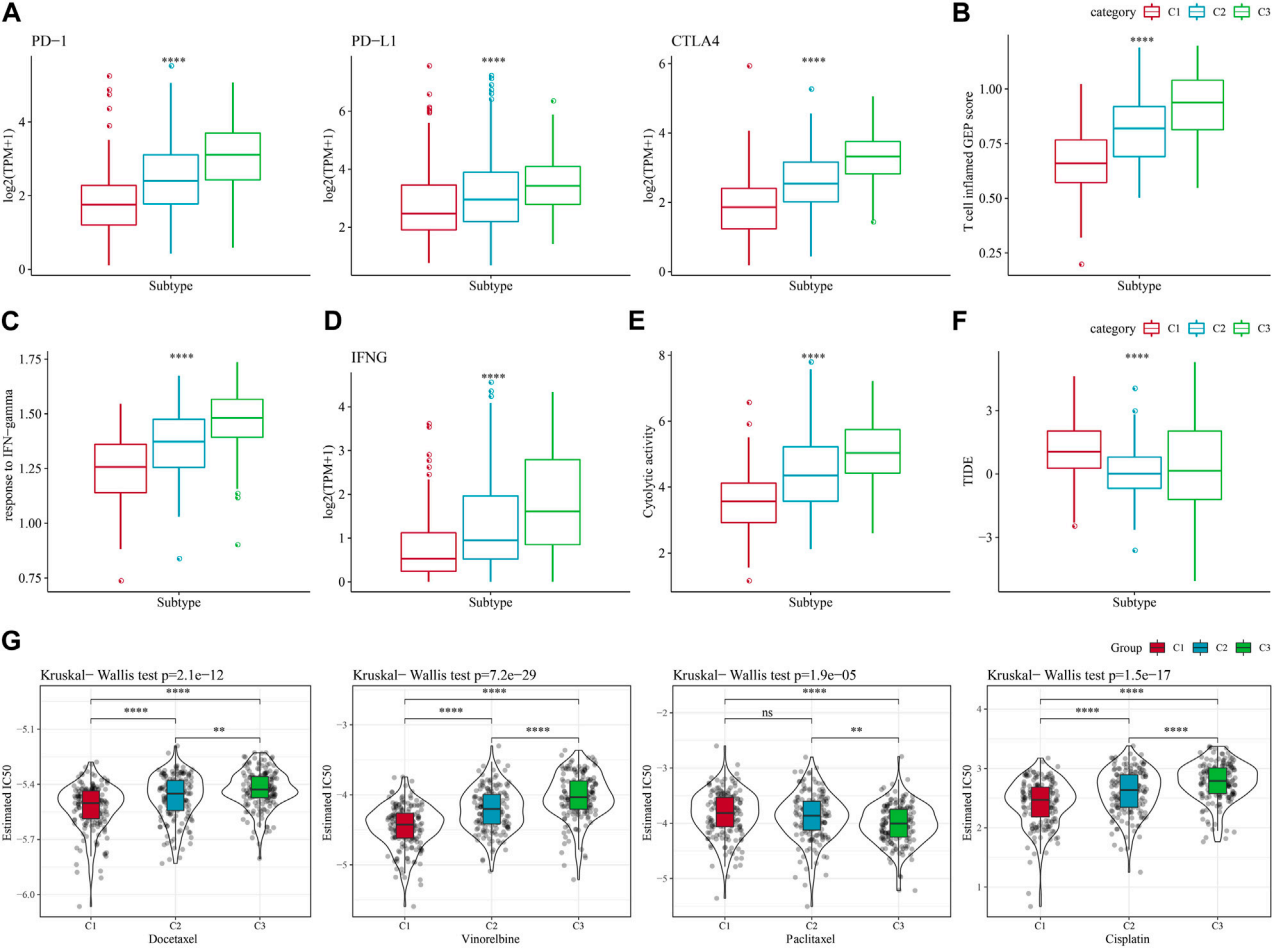
Differences in immunotherapy/chemotherapy treatment between molecular subtypes. **(A)** Differences in expression of immune checkpoint genes between molecular subtypes. **(B)** difference in “T cell inflamed GEP score” between molecular subtypes. **(C)** Differences in “response to IFN-gamma” between different molecular subtypes. **(D)** Differences in INFG gene expression in different isoforms. **(E)** Differences in “Cytolytic activity” between different molecular subtypes. **(F)** Differences in TIDE scores between subtypes. **(G)** Box plots of estimated IC50 of docetaxel, vincristine, paclitaxel and cisplatin in TCGA-LUAD.

In recent years, immunotherapy has led to new opportunities in the treatment of small cell lung cancer. Clinical trials of some immune checkpoint inhibitors have demonstrated their efficacy and safety in LUAD (Hua et al., 2021). Based on this, we first evaluated the expression of the representative molecules of immunotherapy (PD-1, PD-L1, CTLA4) among the three molecular subtypes, and observed that PD-1, PD-L1, and CTLA4 were significantly more expressed in C3 subtype (*p* < 0.05) (Figure 5A). We also applied the “T-cell-inflamed GEP score” to assess the predictive potential of different molecular subtypes to cancer immunotherapy, and the results also showed that the score was highest in C3 (Figure 5B). Considering that IFN-γ is a cytokine that plays a key role in immunomodulation and immunotherapy, we downloaded the GOBP_RESPONSE_TO_INTERFERON_GAMMA gene set from the GO database for ssGSEA analysis, and found that the IFN-γ response was significantly enhanced in the C1 subtype (Figure 5C). We also compared the differences in INFG gene expression in the three subtypes and found that INFG was noticeably high-expressed in the C3 subtype (Figure 5D). Moreover, CYT score, which reflects the cytotoxic effect, was significantly higher in the C3 subtype than in the other subtypes (Figure 5E). In addition, the TIDE prediction data indicated that the C1 subtype had a higher TIDE score, suggesting that the C1 subtype was less likely to benefit from immunotherapy (Figure 5F). The estimated IC50 of docetaxel, vincristine, paclitaxel and cisplatin among 3 subtypes showed that C1 was more sensitive to the four chemotherapy drugs (Figure 5G). The above results indicated that predicting immunotherapy for LUAD based on NK cell-related genes was a practical approach.

### 3.6 The analysis of PCD patterns among molecular subtypes

**FIGURE 6:**
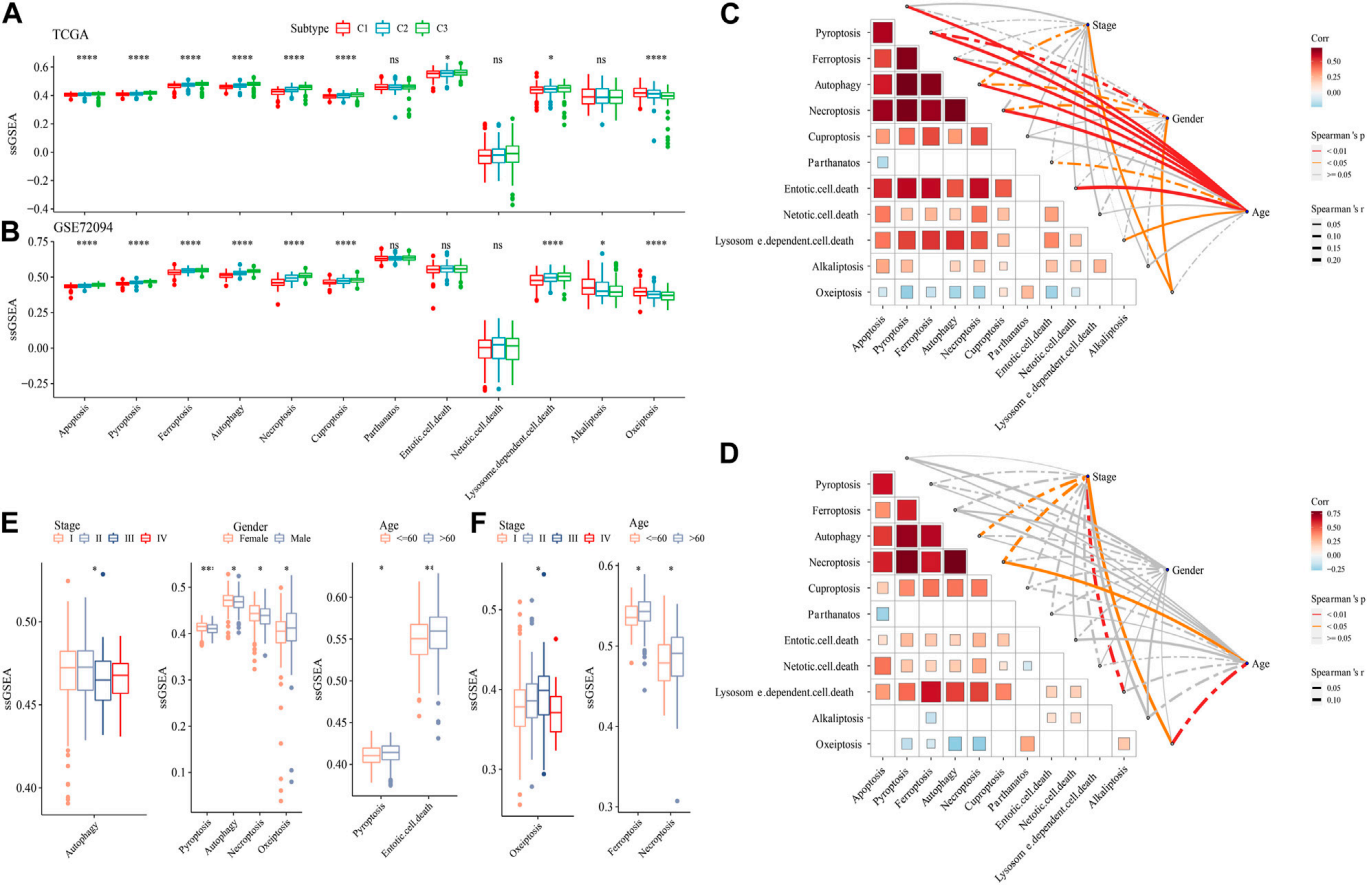
The PCD characteristic among 3 molecular subtypes. **(A)** the ssGSEA analysis of 12 PCD patterns among 3 molecular subtypes in TCGA-LUAD dataset. **(B)** the ssGSEA analysis of 12 PCD patterns among three molecular subtypes in GSE72094 dataset. **(C)** The spearman analysis between clinical feature and PCD in TCGA-LUAD dataset. **(D)** The spearman analysis between clinical feature and PCD in GSE72094 dataset. **(E)** The ssGSEA analysis of PCD in TCGA-LUAD samples with Stage, Gender and Age. **(F)** The ssGSEA analysis of PCD in GSE72094 samples with Stage, and Age.

The ssGSEA analysis calculated the score of 12 PCD patterns in each sample in TCGA dataset and GSE72094 dataset. We found that 9 PCD scores had differences among 3 subtypes in both two datasets (Figures 6A, B). In TCGA dataset, Stage, Gender, especially, Age had closely associated to PCD patterns (Figure 6C), but in GSE72094 dataset, clinical features had litter associated to PCD patterns (Figure 6D). Autophagy score were increased in early Stage, the scores of Pyroptosis, Autophagy, Necroptosis and Oxeiptosis were enhanced in Male samples, and samples with age > 60 had higher Pyroptosis, Entotic. cell.death scores in TCGA dataset (Figure 6E). In GSE72094 dataset, Oxeiptosis score was highest in StageⅢ, and Ferroptosis and Necroptosis scores were greater in patients with age>60 (Figure 6F).

### 3.7 Establishment of LUAD risk model

We first calculated the NK cell-related genes significantly differentially expressed among the three molecular subtypes by the limma package, significant expression differences of NK cell-related genes among C1, C2, and C3 were detected, including 11 upregulated genes and 180 downregulated genes (Supplementary Figures S1A, B). Differentially expressed downregulated genes were related to immune-related pathways, as shown by the results of enrichment analysis (Supplementary Figure S1C). Genes with upregulated level were related to inflammatory and immune pathways (Supplementary Figure S1D). 173 genes with high prognostic impact (*p* < 0.05), including 159“Protective” and 14“Risk” genes, were identified from those genes by conducting one-way Cox regression analysis (Supplementary Figure S2A). Further, we observed the trajectory of each gene with lambda using Lasso analysis, and the model was optimal when lambda = 0.0382, which corresponded to 9 differential genes (Supplementary Figures S2B, C). After that, we reduced the genes to four, namely, ANLN, FAM83A, RHOV, and PARP15, by the stepAIC method in the MASS package (Supplementary Figure S2D).

**FIGURE 7:**
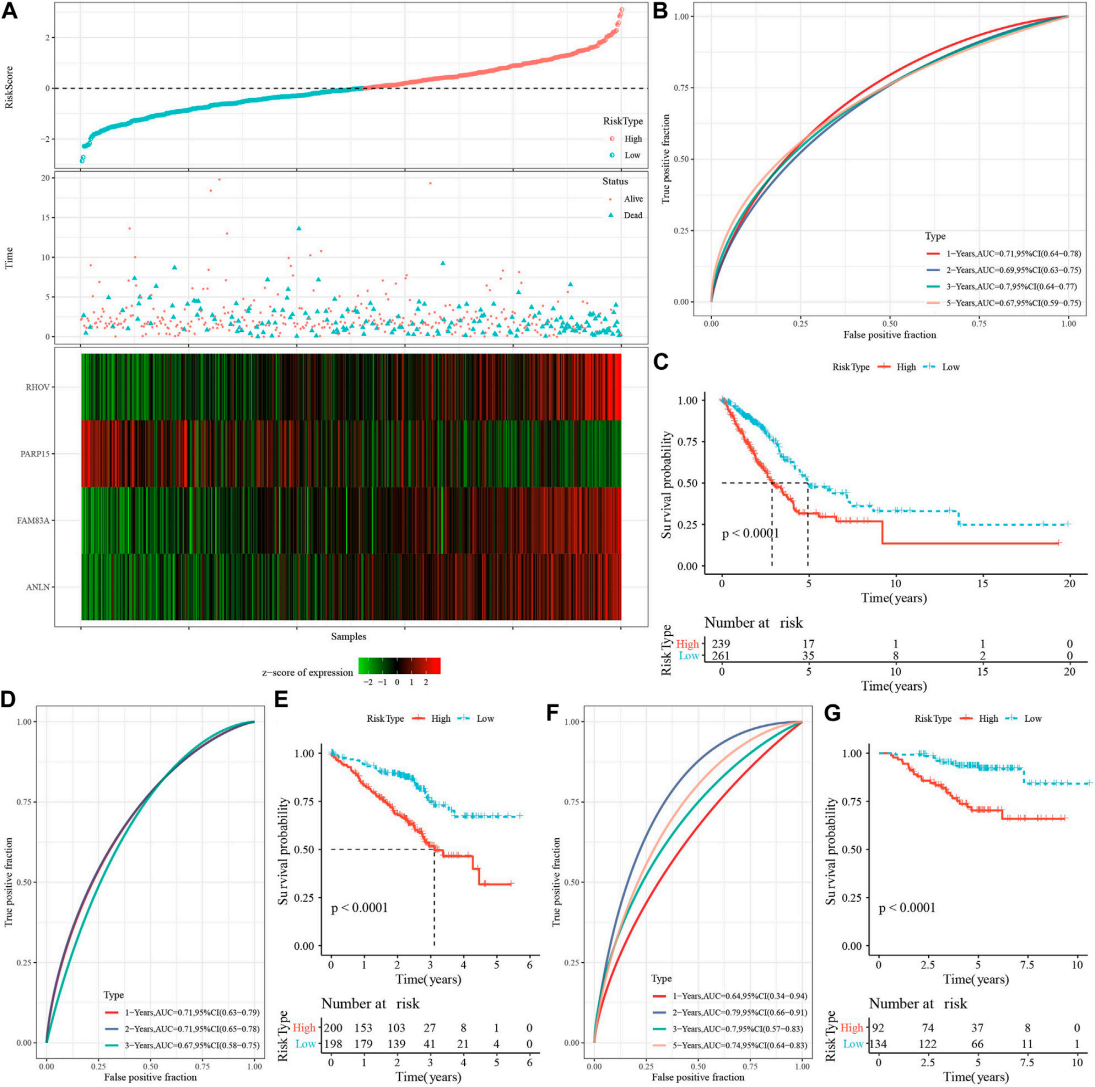
Risk modeling and validation. **(A)** RiskScore, survival time vs. survival status and expression of necroptosis-related genes in TCGA-LUAD dataset. **(B)** ROC curves with AUC for RiskScore classification in the TCGA-LUAD dataset. **(C)** Distribution of KM survival curves for RiskScore in the TCGA-LUAD dataset. **(D,E)**: ROC curves and KM survival curves for RiskScore in the GSE72094 cohort. **(F,G)**: ROC curves and KM survival curves of RiskScore in the GSE31210 cohort.

Then, we calculated the Riskscore score for each TCGA-LUAD patient using these four genes and the above formula (Figure 7A). We classified those RiskScore with 0 ≤ as low-risk group and with RiskScore >0 as high-risk group. Then, we performed a prognostic classification ROC analysis in the “timeROC” package for analyzing 1-year, 2-year, 3-year, and 5-year prognostic prediction classification efficiency, and we found that the model had a high AUC (0.71, 0.69, 0.7, and 0.67) (Figure 7B). The results of survival analysis showed that patients in the low-risk group developed a significantly better prognosis (*p* < 0.001) (Figure 7C). To confirm the robustness of this clinical prognostic model, we validated it in the GSE72094 and GSE31210 cohorts and used the same approach to calculate the RiskScore of patients (Figures 7D–G).

### 3.8 Pathological characteristics of high- and low-risk groups

**FIGURE 8:**
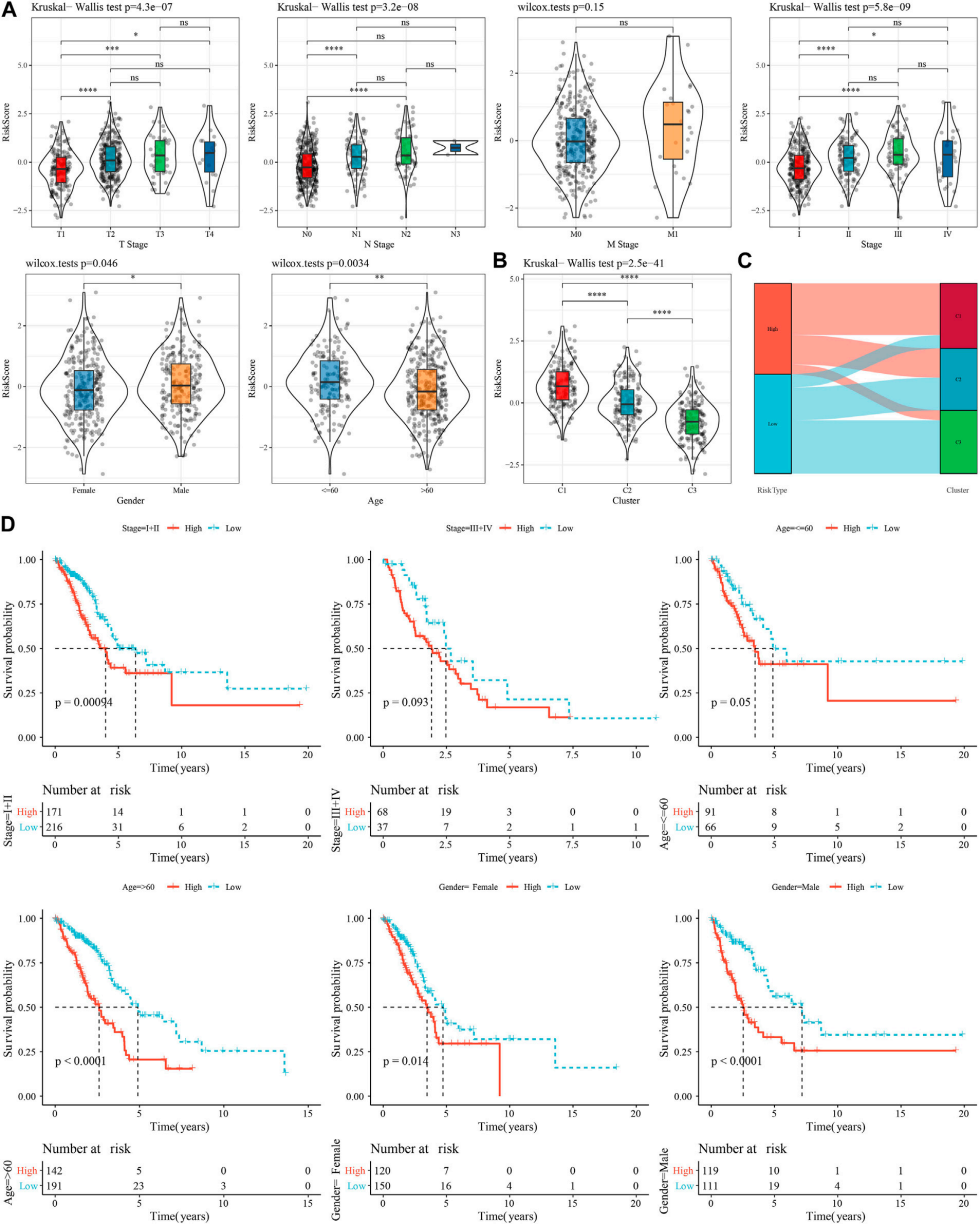
Performance of RiskScore in TCGA-LUAD cohort with different clinicopathological characteristics. **(A)** Differences between RiskScore between different clinicopathological subgroups in the TCGA-LUAD cohort. **(B)** Differences in RiskScore between different molecular subtypes in the TCGA-LUAD cohort. **(C)** Differences between molecular subtypes and RiskScore subgroups in the TCGA-LUAD cohort. **(D)** KM curves between high- and low-risk groups in the TCGA-LUAD cohort between different clinicopathological subgroups.

To investigate the reliability of this risk model classification method, we first compared the clinical characteristics of patients in both high- and low-risk groups. The results showed that the RiskScore scores of patients with Stage III-IV, M Stage, N Stage, T Stage were significantly higher than Stage I-II ones. In addition, we also found that male patients had a higher RiskScore (Figure 8A). Also, we compared the differences in RiskScore by molecular subtype and found that the RiskScore for the C1 subtype with poorer prognosis was significantly higher than C3 with a better prognostic outcome (Figure 8B), and that the majority of the samples with high RiskScore were “C1” patients (Figure 8C). In addition, we also compared whether there was a prognostic difference in the—high- and low-risk groups between the different clinicopathological characteristics subgroups in the TCGA-LUAD cohort. Across different clinical subgroups, the risk grouping performed equally well, pointing to the reliability of the grouping (Figure 8D). This finding also applied to the GSE72094-LUAD cohort (Supplementary Figure S3).

### 3.9 Immune infiltration and pathway characteristics of low-risk and high-risk patients

**FIGURE 9:**
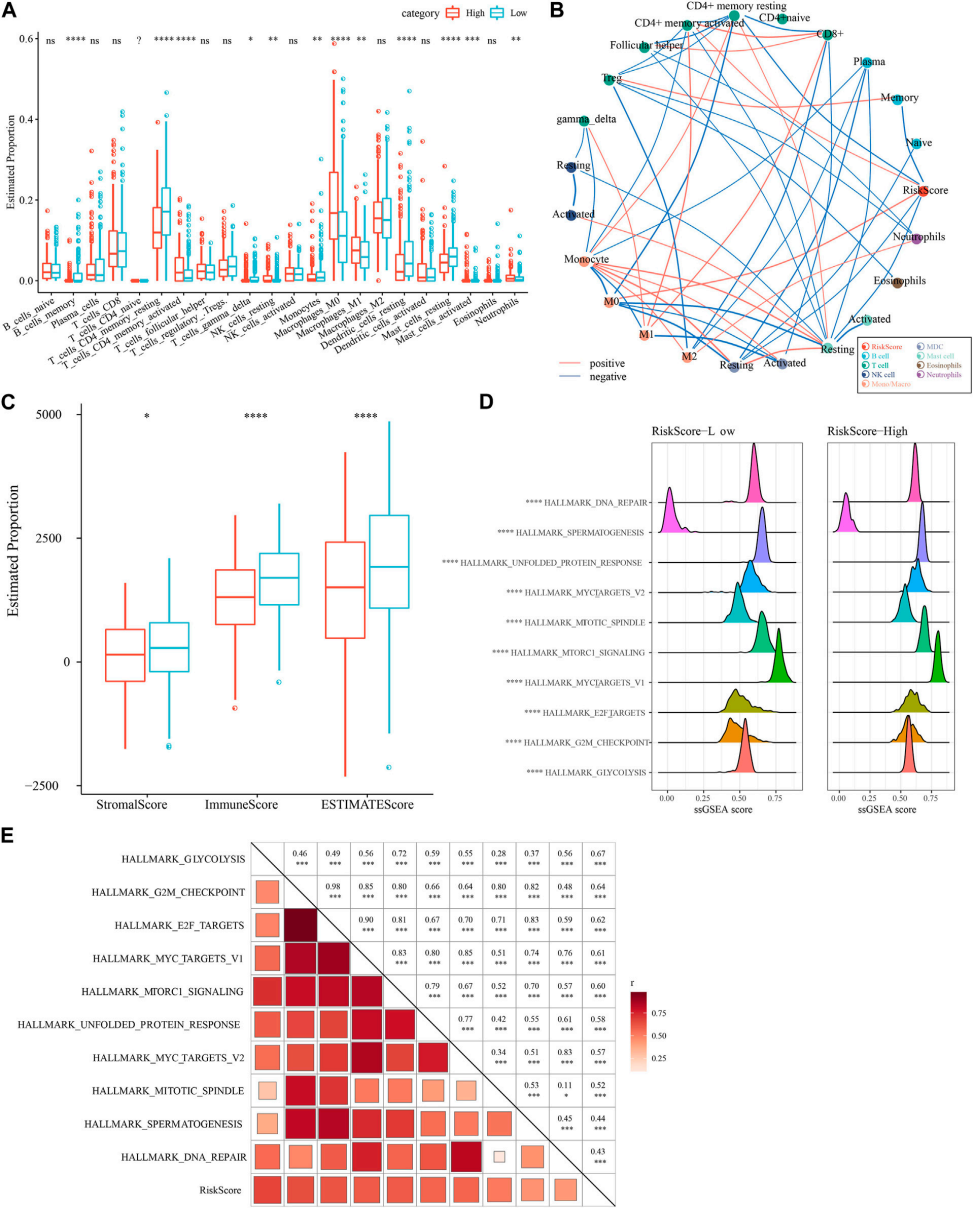
Immune infiltration/pathway characteristics between RiskScore subgroups. **(A)** Proportion of immune cell components in the TCGA cohort. **(B)** Correlation analysis of 22 immune cell components in the TCGA cohort with the RiskScore. **(C)** Proportion of immune cell components in the TCGA cohort calculated by ESTIMATE software. **(D)** The top 10 pathways with the most significant differences between high- and low-groups. **(E)** Results of correlation analysis between the RiskScore and KEGG pathways with correlation greater than 0.4.

We compared the relative abundance of 22 immune cell types in the two subgroups of the TCGA-LUAD cohort in high- and low-risk groups. We discovered that the majority of immune cells (B cells, macrophages, T cells, and mast cells) were significantly different in high- and low-risk groups (*p* 0.05, Figure 9A). It is worth noting that activated NK cells had no significance between high- and low-group. We also examined the connection between the RiskScore and 22 immune cell components (Figure 9B). Also, we assessed the immune cell infiltration using the ESTIMATE method. The three scores were significantly different between two risk groups (*p* < 0.05), and the low-Riskscore group had higher immune infiltration (Figure 9C). The relationship between biological function in different samples with RiskScore was analyzed by “ssGSEA” analysis and found that the high risk group was significantly enriched to some cell cycle-related pathways, such as HALLMARK_SPERMATOGENESIS, and HALLMARK_REPAIR, SPERMATOGENESIS, HALLMARK_DNA_REPAIR, ALLMARK_MYC_TARGETS_V2, HALLMARK_UNFOLDED_PROTEIN_RESPONSE, *etc.* (Figure 9D). Further, we selected functional pathways with correlations greater than 0.4, from which we could see that RiskScore showed positive correlation with cell cycle-related pathways, such as HALLMARK_MYC_TARGETS_V1, HALLMARK_MTORC1_SIGNALING, HALLMARK_E2F_TARGETS (Figure 9E).

### 3.10 Differences in immunotherapy/chemotherapy for patients in high- and low-risk groups

**FIGURE 10:**
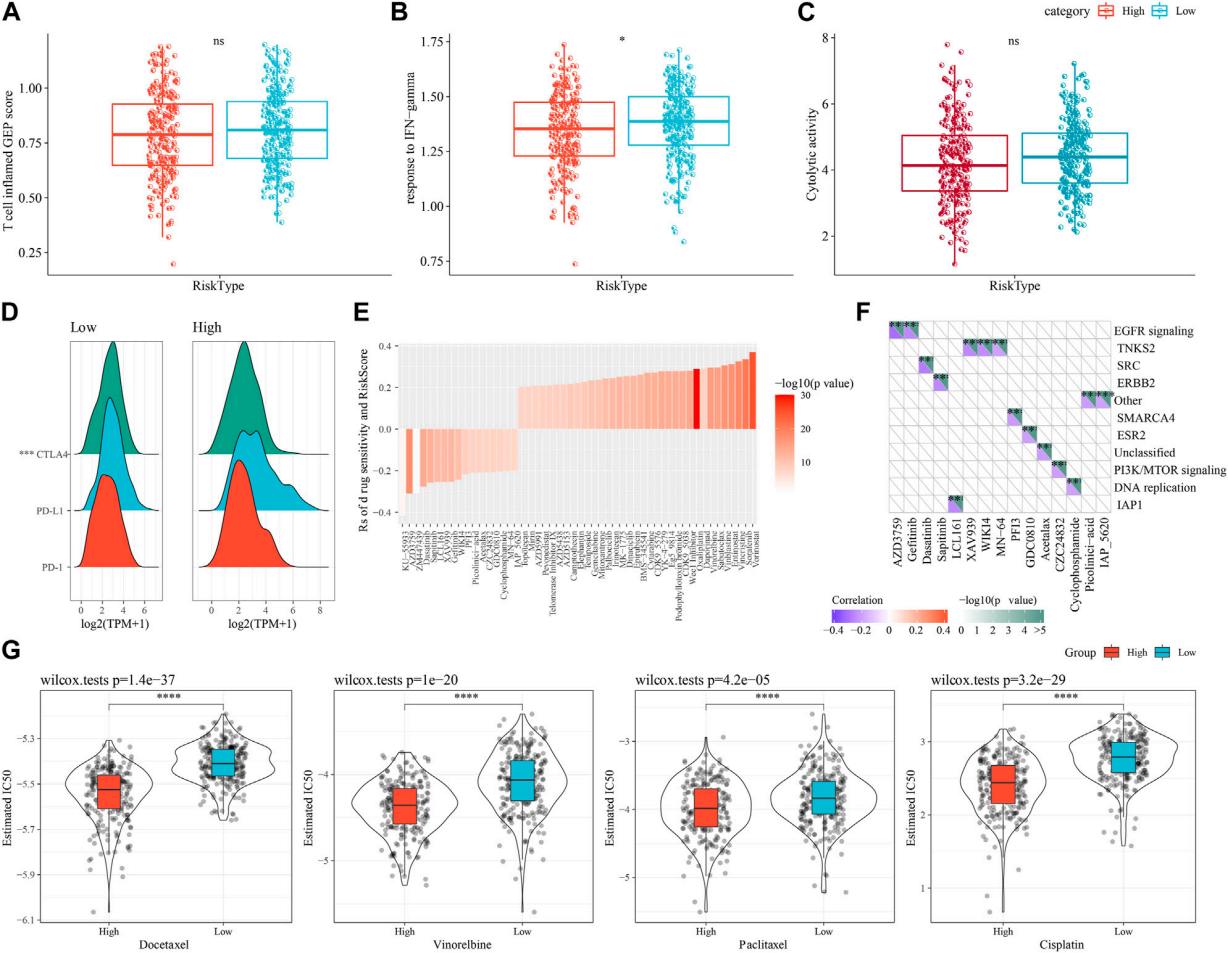
Differences in immunotherapy/chemotherapy between RiskScore subgroups. **(A)** Difference in “T cell inflamed GEP score” between molecular subtypes. **(B)** Difference in “response to IFN-γ" between molecular subtypes. **(C)** Differences in “Cytolytic activity” between molecular subtypes. **(D)** Differences in expression of immune checkpoint genes between molecular subtypes. **(E)** 15 pairs drugs were significantly associated to RiskScore. **(F)** 15 pairs drugs mainly target EGFR signaling and TNKS2 pathways. **(G)** IC50 box plots of docetaxel, vincristine, paclitaxel and cisplatin in TCGA-LUAD dataset.

First, we used the “T-cell-inflamed GEP score” to assess the predictive potential of the different RiskScore subgroups in cancer immunotherapy. The results showed that the “T-cell-inflamed GEP score” was elevated in the low-risk group, but the difference was not statistically significant (Figure 10A), however, in the low-risk group the IFN-γ response was noticeably elevated (Figure 10B). The CYT score, which reflects cytotoxic effects, was elevated in the low-risk group, showed no statistically significant differences (Figure 10C). The expression of representative molecules of immunotherapy (CTLA4, PD-L1, and PD-1) was calculated in the risk groups and showed that CTLA4 was significantly more expressed in the low-risk group (*p* < 0.05), while the difference in PD-1 and PD-L1 expression was not significant (Figure 10D). We looked at the connection between RiskScore and medication response in cancer cell lines to better understand the impact of RiskScore on drug response. We found 49 substantially linked relationships between RiskScore and drug sensitivity in the Genomics of Drug Sensitivity in Cancer (GDSC, http://cancer.sanger.ac.uk/cell_lines#) database using Spearman correlation analysis. Of these 49 pairs, 15 pairs were significantly associated with Riskscore correlations, such as Vinorelbine, Sabutoclax, Vinblastine, Entinostat, Vincristine, and Sorafenib (Figure 10E). We found that these drugs mainly target the EGFR signaling and TNKS2 pathways through the study on the signaling pathways of the genes targeted by these drugs (Figure 10F). In addition, we also explored the response of different molecular subtypes in the TCGA-LUAD cohort to the traditional chemotherapeutic agents Docetaxel, Vinorelbine, Paclitaxel and Cisplatin, and found that overall patients in the high-risk group were more sensitive to all the four chemotherapeutic agents (Figure 10G), suggesting that patients in the high-risk group may benefit from these four drugs.

### 3.11 PCD characteristics in high- and low-risk groups

**FIGURE 11:**
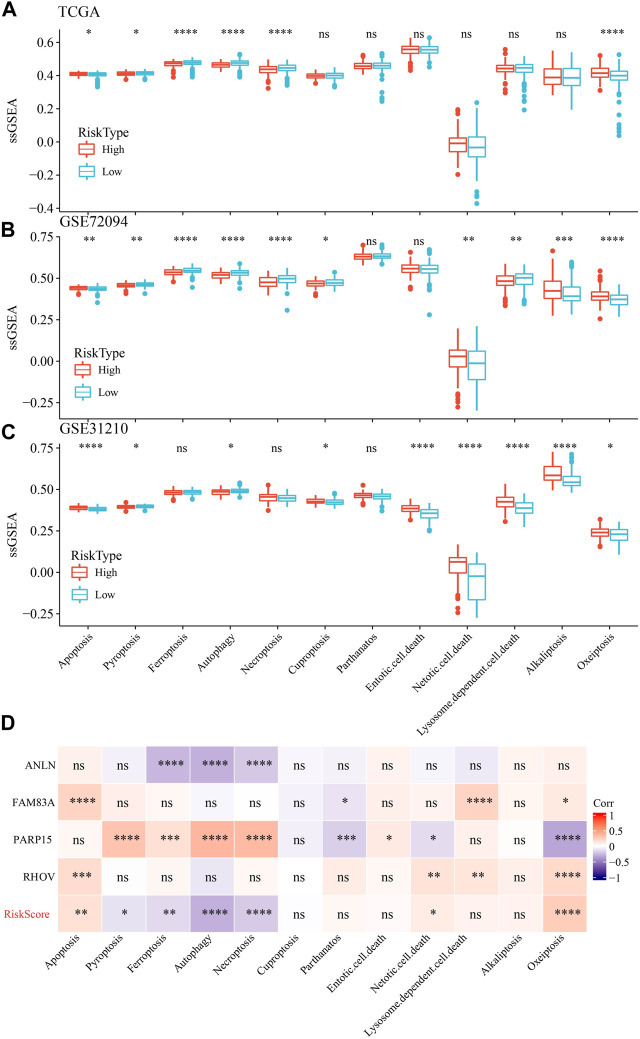
The ssGSEA analysis of 12 PCD patterns in high- and low-groups. **(A)** ssGSEA analysis of 12 PCD in high- and low-group in TCGA-LUAD dataset. **(B)** ssGSEA analysis of 12 PCD in high- and low-group in GSE72094 dataset. **(C)** ssGSEA analysis of 12 PCD in high- and low-group in GSE31210 dataset. **(D)** the association among RiskScore, model genes and 12 PCD patterns.

We also determine the PCD characteristics in high- and low-risk groups using ssGSEA analysis. 6 of 12 PCD styles had differences between high- and low-risk groups in TCGA dataset (Figure 11A). In GSE72094 dataset, 10 PCD patterns scores presented differentiation in high- and low-risk groups (Figure 11B). Moreover, the differences of 9 PCD scores between high- and low-groups was observed in GSE31210 dataset (Figure 11C). RiskScore as well as four model genes were obviously related to PCD patterns (Figure 11D).

### 3.12 RiskScore combined with clinicopathological features to further improve prognostic models and survival prediction

**FIGURE 12:**
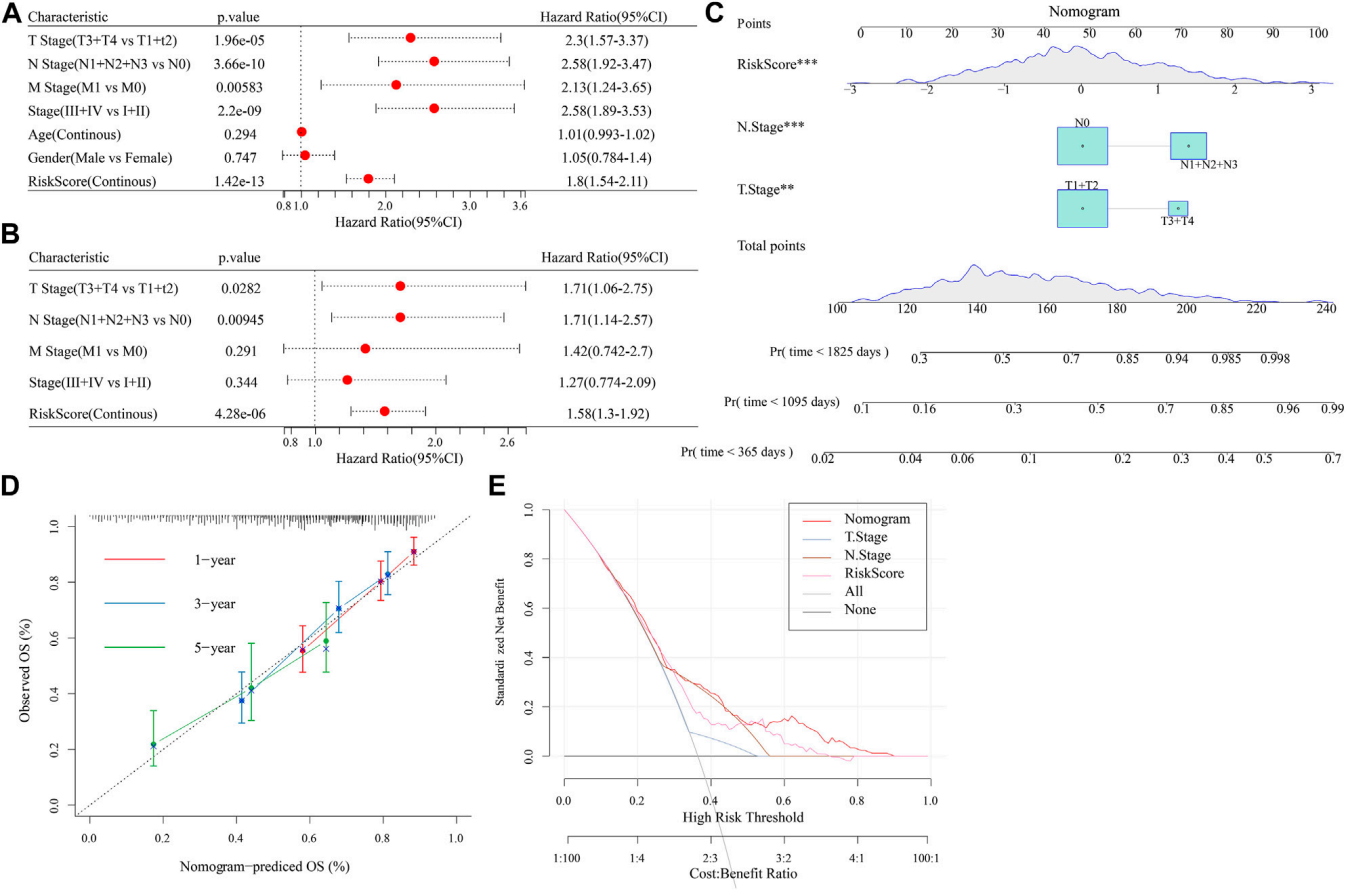
Establishment of nomogram. **(A,B)**: Univariate and Multivariate Cox analysis of RiskScore and clinicopathological characteristics; **(C)**: The nomogram model; **(D)**: Calibration curves for 1, 3, and 5 years for the nomogram; **(E)**: Decision curves for the nomogram.

Univariate and multifactorial Cox regression analyses revealed RiskScore as the most significant prognostic factor (Figures 12A, B). We created a nomogram (Figure 12C) combining RiskScore and other clinicopathological traits for the risk assessment and prediction of survival probability for LUAD patients. The model results revealed that RiskScore had the biggest influence on survival prediction. The prediction calibration curves at the three calibration points of 1, 3, and 5 year(s) nearly overlapped with the standard curve, which indicated that the nomogram plot had excellent prediction performance. We further assessed the prediction accuracy of the model using the calibration curve (Figure 12D). We also used DCA (Decision curve) to test the model’s dependability, and it was shown that RiskScore and Nomogram performed much better than the extreme curve and had the strongest ability to predict survival among other clinicopathological factors (Figure 12E).

## 4 Discussion

Lung cancer is currently the most aggressive malignancy in the world, of which LUAD is the most common histological subtype of primary lung cancer, accounting for 64% of peripheral lung cancers, and has been reclassified from invasive precancerous lesions to invasive adenocarcinoma (Denisenko et al., 2018; Hutchinson et al., 2019). Despite the current advances in the treatment of LUAD, the median survival is only 8.6 months and immune escape is considered one of the main factors leading to treatment failure in LUAD (Yotsukura et al., 2021). In contrast to the remarkable efficacy of immune checkpoint inhibitor (ICI) in metastatic melanoma, Hodgkin’s lymphoma, and bladder cancer, not all patients with LUAD are sensitive to ICI (Zhang et al., 2020). Mechanisms of immune escape that lack adaptive immune response include hypoxia-driven immunosuppressive factors, anti-apoptotic pathways, chronic inflammation, metabolic damage, and immune cells such as regulatory T (Treg) cells, tumor-associated M2 macrophages (TAM), myeloid-derived suppressor cells (MDSC) (Yu et al., 2021). Recent studies have shown that T and NK cell dysfunction and depletion or deficiency of antitumor-specific effector cells are involved in LUAD immune escape (Hong et al., 2019), and although the exact mechanism is unclear, it points to new ideas for the study of immune escape in LUAD and provides new targets for immunotherapy in LUAD.

LUAD is usually resistant to chemotherapy and/or radiotherapy and leads to the development of distant metastases (Jiang et al., 2021). NK cell dysfunction and failure in patients with LUAD could be caused by immune escape mechanisms mediated by lung cancer cells or tumor microenvironment, leading to failure of immunotherapy. The reason for this is related to tumor upregulation of inhibitory ligands (e.g., HLA-C molecules) and recognition by autoinhibitory KIR receptors carrying ITIM motifs (Daëron et al., 2008). Cellular experiments showed that other inhibitory receptors, for instance, KLRG-1, LAG-3, CD94/NKG2A, TIM3, TIGIT, and their ligands were also frequently upregulated on NK cells from LUAD patients (Lee et al., 1998; Nayyar et al., 2019), which was consistent with our study, where we found significantly different NK cell-related gene expression in different subtypes. CTLA-4 (ipilimumab) improved clinical prognosis of patients with LUAD (Paulsen et al., 2017) in addition to the common PDL-1 inhibitors (avelumab, atezolizumab, durvalumab) and PD-1 (camrelizumab, spartalizumab, nivolumab, pembrolizumab). Our study identified the expression patterns of PD-1/PD-L1 and CTLA-4 in different subtypes, confirming a possible immune escape mechanism of NK cells in LUAD and providing a new perspective for blocking immune dysregulation.

The tumor microenvironment (TME) consists of associated fibroblasts (CAF), tumor cells, other immune cells, and endothelial cell constituents (ECs) (Vitale et al., 2019). Ghiringhelli F et al. showed that suppressive immune cells such as Treg cells, CTLA-4+ regulatory, and that N2 neutrophils and M2 macrophages can disrupt the anti-lung cancer activity of NK cells (Domagala-Kulawik et al., 2014). Similarly, our data showed significant differences in the proportion of NK cells, B cells, and T cell content between different molecular subtypes, suggesting that other immune cells may impair the cytotoxic and migratory activity of NK cells with numerical and functional advantages, and thus causing NK cell depletion (Bi and Tian, 2017). But we found that activated NK cells had no differences between high- and low-group, maybe caused by insufficient samples.

Changes in NK cell counts, including peripheral blood, circulation and TME in healthy individuals, can be used as prognostic markers in patients with head and neck and lung tumors (Lin et al., 2017; Lin et al., 2020; Zhong et al., 2021). We constructed the prognosis model by NK cell-related genes (ANLN, FAM83A, RHOV, and PARP15), which is a powerful tool to assist clinical decision-making with effective prediction of patient survival and drug sensitivity. ANLN is an actin-binding protein, and previous studies have demonstrated that ANLN is associated with actin cytoskeleton dynamics (Xu et al., 2019). Xu J et al. showed that ANLN overexpression promotes distant metastasis of lung cancer cells and is associated with epithelial mesenchymal transformation (EMT) of LUAD cells transformation (EMT) in LUAD cells. Similar to previous bioinformatic analyses, our study found that upregulated FAM83A in LUAD tissues, which was relate to LUAD prognosis (Suzuki et al., 2005; Deng et al., 2021). Knockdown of FAM83A inhibited proliferation, migration and invasion of LUAD cells. In addition, the lncRNA FAM83A-AS1 regulates FAM83A expression by acting as a competing endogenous RNA for miR-495-3p (Wang et al., 2021). These results suggested that FAM83A plays an oncogenic role in LUAD and that FAM831-AS1 can regulate FAM83 expression by taking up miR-495-3p. Similar to FAM83A, invasion, migration and proliferation of LUAD cells could be stimulated by RHOV overexpression, while knockdown of RHOV inhibits the functionalistic behavior of the cells. In addition, RHOV knockdown inhibits metastasis and LUAD tumor growth of nude mice, which may be related to RHOV activation of the JNK/c-Jun signaling pathway (Zhang et al., 2021c). There are fewer basic studies on PARP15 in LUAD, and genomic data with large sample sizes suggested that RHOV is a useful marker for immunotherapy and survival in LUAD (Han et al., 2020). The above studies revealed a novel regulatory mechanism of NK cells in LUAD tumor development, which may be a new biomarker and therapeutic target for LUAD.

Docetaxel, Vinorelbine, Paclitaxel and Cisplatin are currently widely used chemotherapy drugs for lung cancer, which cause cell cycle arrest (Clegg et al., 2001; Dasari and Tchounwou, 2014). However, resistance can develop, leading to further tumor development and side effects such as myelosuppression, drug nephritis, nausea, vomiting, hearing loss and polyneuropathy, which will significantly reduce the patient’s quality of life (Dasari and Tchounwou, 2014). Acquired chemotherapy resistance is a major problem faced by clinicians and a major cause of treatment failure. Regardless of the type of resistance, loss of tumor sensitivity to the drug leaves very little time for therapy to correct, with the goal of improving patient survival. Patients’ clinical outcomes can be significantly improved by personalizing treatment regimens and predicting the effects of drug therapy. The results of this study showed that patients in C1 subtype and high-risk group were more sensitive to and benefited from four chemotherapy drugs. We speculated that may be the number of NK cells affects drug sensitivity.

Although this study reveals the immune signature of NK cell-related genes in LUAD and confirms the role in prognosis and immunotherapy of LUAD, the following limitations remain: (Hirsch et al., 2017): The wide variety, rapid development of bioinformatics tools can help predict potential key molecules and pathways, narrow the scope and improve the efficiency of the study, but the final findings should be validated based on real genetic data in basic and clinical settings; (Siegel et al., 2022); The database used to conduct functional and signaling pathway enrichment analysis has comprehensive and complete data, but its slow updates may have some unpredictable effects on the results; (Succony et al., 2021); The results were based on extrapolation of the raw signal algorithm and should be supported by further laboratory and clinical evidence.

## 5 Conclusion

Based on NK cell-related genes, we identified three stable molecular subtypes of LUAD, which differed significantly in terms of immunity, pathways, prognosis and drug sensitivity among different molecular subtypes. Based on NK cell-related genes, this study developed a prognostic model, which was highly robust and had a greater potential for application in predicting immunotherapeutic response and patient prognosis.

## Data Availability

The original contributions presented in the study are included in the article/Supplementary Materials, further inquiries can be directed to the corresponding author.
