# Supplementary figures and images for: Identification of natural killer cell associated subtyping and gene signature to predict prognosis and drug sensitivity of lung adenocarcinoma
Source: Front Genet. 2023 Apr 7;14:1156230. doi: 10.3389/fgene.2023.1156230 (PMC10119412; doi:10.3389/fgene.2023.1156230)

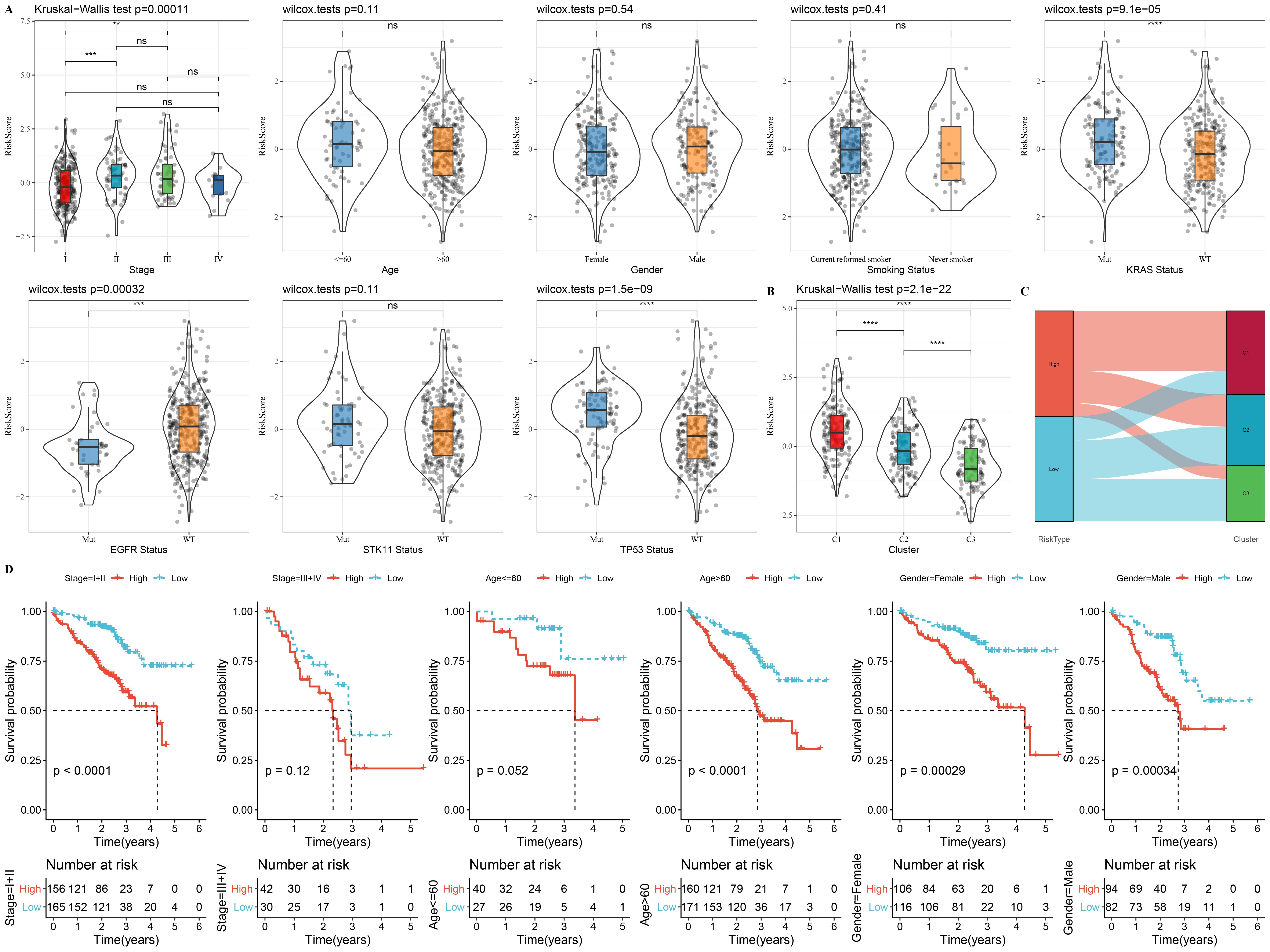

Supplement: Supplementary file 1 [file Image3.JPEG]

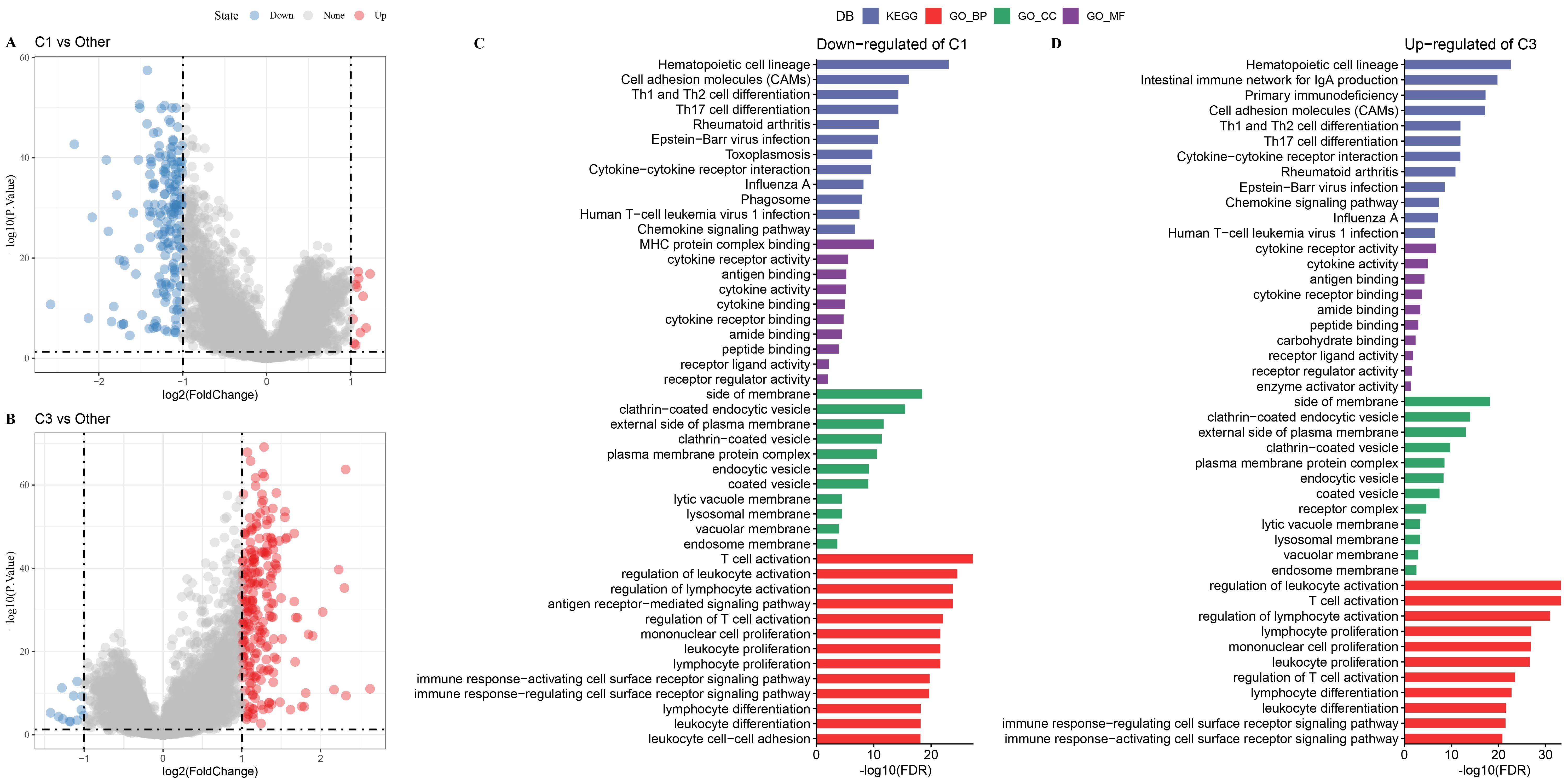

Supplement: Supplementary file 2 [file Image1.JPEG]

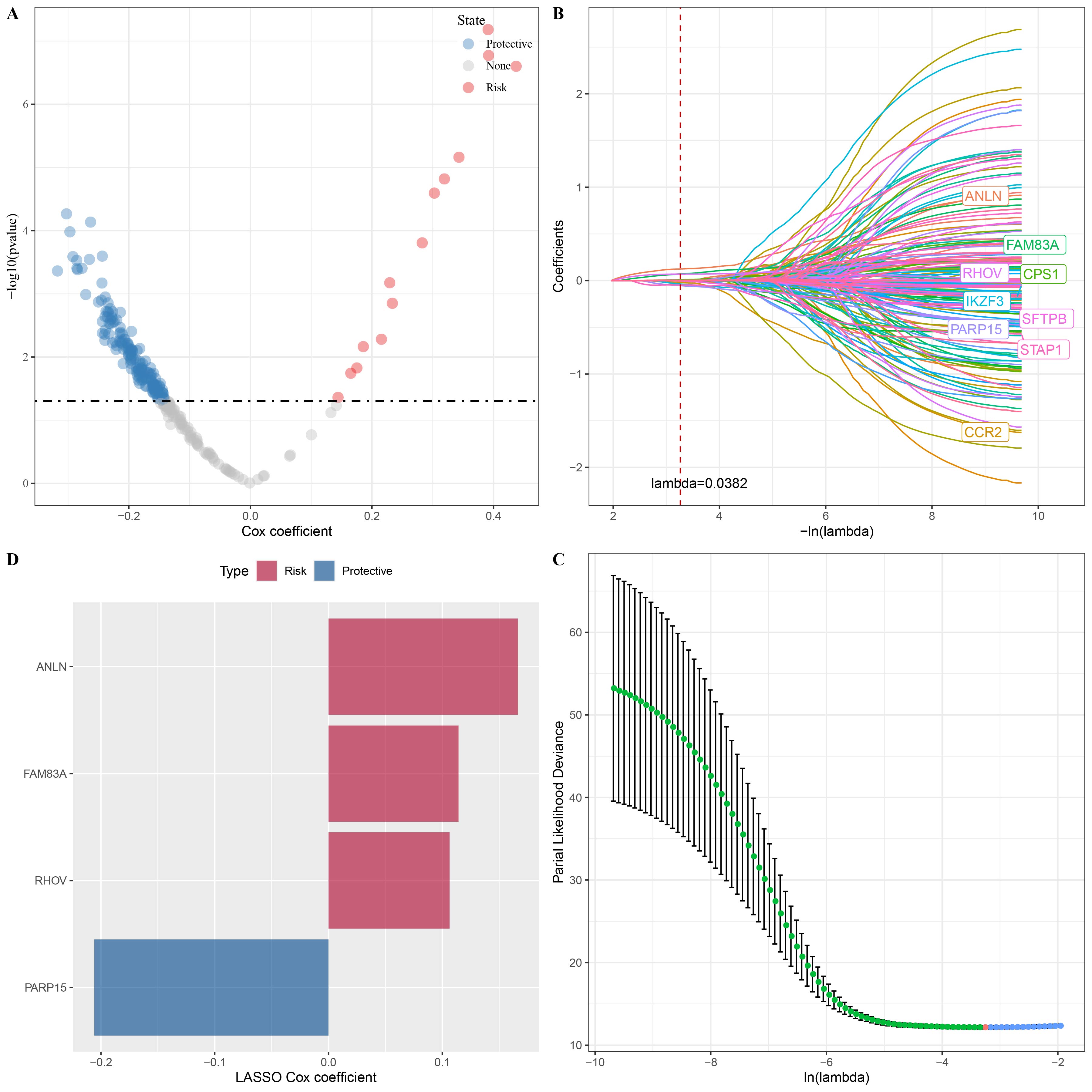

Supplement: Supplementary file 3 [file Image2.JPEG]
