# Supplementary material for: Identification of natural killer cell associated subtyping and gene signature to predict prognosis and drug sensitivity of lung adenocarcinoma
Source: Front Genet. 2023 Apr 7;14:1156230. doi: 10.3389/fgene.2023.1156230 (PMC10119412; doi:10.3389/fgene.2023.1156230)
Supplement: Supplementary file 4 [file DataSheet2.docx]

The data analyzed in this study:

https://www.jianguoyun.com/p/DTgNSaEQ5oytCxjwlfUEIAA
