# Supplementary material for: Identification of natural killer cell associated subtyping and gene signature to predict prognosis and drug sensitivity of lung adenocarcinoma
Source: Front Genet. 2023 Apr 7;14:1156230. doi: 10.3389/fgene.2023.1156230 (PMC10119412; doi:10.3389/fgene.2023.1156230)
Supplement: Supplementary file 5 [file DataSheet1.docx]

**Supplementary material description**

**Supplementary Figure S1** Differential gene expression analysis between molecular subtypes. A: Volcano plot of C1 vs Other differentially expressed genes in the TCGA-LUAD cohort. B: Volcano plot of C3 vs Other differentially expressed genes in the TCGA-LUAD cohort. C: Results of GO, KEGG functional enrichment analysis of C1 vs Other differentially down-regulated genes in the TCGA-LUAD cohort.D:Results of GO, KEGG functional enrichment analysis of C3 vs Other differentially upregulated genes in the TCGA-LUAD cohort.

**Supplementary Figure S2** Identification of key genes in NK cells. A: A total of 173 potential genes were identified among the DEGs; B: Trajectory of each independent variable with lambda.C: Confidence intervals under lambda; D: Distribution of LASSO coefficients for natural killer cell-associated gene features.

**Supplementary Figure S3** Performance of RiskScore in GEO-LUAD cohort with different clinicopathological characteristics. A: Differences of RiskScore between different clinicopathological subgroups in the GEO-LUAD cohort. B: Differences of RiskScore between different molecular subtypes in the GEO--LUAD cohort. C: Differences between molecular subtypes and RiskScore subgroups in the GEO--LUAD cohort; D: KM curves between riskScore-high and -low groups in the GEO-LUAD cohort between different clinicopathological subgroups.
